# Interpretable survival modeling integrating nutritional-inflammatory biomarkers in elderly patients with locally advanced esophageal squamous cell carcinoma treated with definitive radiotherapy

**DOI:** 10.3389/fimmu.2026.1836567

**Published:** 2026-05-11

**Authors:** Jie Zou, Wenjing Fan, Xingzhuo Xia, Mengyue Zhang, Tingting Han, Qibing Wu

**Affiliations:** 1 Department of Radiology Oncology, The First Affiliated Hospital of Anhui Medical University, Hefei, Anhui, China; 2 Department of Radiation Oncology, The First Hospital of the University of Science and Technology of China, Hefei, Anhui, China

**Keywords:** definitive radiotherapy, elderly patients, esophageal squamous cell carcinoma, interpretable prognostic modeling, nutritional-inflammatory biomarkers

## Abstract

**Background:**

Elderly patients with locally advanced esophageal squamous cell carcinoma (ESCC) receiving definitive radiotherapy show marked survival heterogeneity. As anatomical staging alone cannot fully reflect host vulnerability, immune-inflammatory condition, and treatment tolerance, interpretable prognostic tools based on routinely available variables may help refine risk assessment in this population.

**Methods:**

This retrospective study enrolled 308 elderly patients with locally advanced ESCC treated with definitive radiotherapy. After excluding 12 patients with missing data, the remaining cases were randomly divided into training and testing cohorts at a 7:3 ratio for internal validation. Nine conventional and machine learning-based survival models were constructed using clinicopathological and nutritional-inflammatory variables, with performance evaluated via concordance index (C-index), time-dependent area under the curve (AUC), and integrated Brier score (IBS). SHAP analysis was performed on top-performing models to clarify predictor contributions over follow-up.

**Results:**

Median overall survival (OS) and progression-free survival (PFS) were 19 and 13 months, respectively. For OS, regularized linear survival modeling showed the most stable performance, with Ridge regression providing the most consistent discrimination in the testing cohort. For PFS, model ranking was more time- and endpoint-dependent, and no complex machine-learning algorithm demonstrated a stable overall advantage. Exploratory age-stratified analyses suggested heterogeneity across elderly subgroups, with different algorithms ranking highest in patients aged 65–74 years and those aged ≥75 years. SHAP analysis showed that nutritional indicators, especially GNRI and PNI, contributed more persistently to OS prediction than to short-term PFS, whereas baseline inflammatory markers such as NLR and SIRI showed relatively weak attribution in this multivariable prediction framework. Ridge-based predicted-risk thresholds identified distinct high- and low-risk groups for both OS and PFS.

**Conclusion:**

In elderly patients with locally advanced ESCC treated with definitive radiotherapy, regularized linear models showed robust prognostic utility, whereas more complex tree-based or boosting models did not demonstrate a stable advantage. Nutritional markers, particularly GNRI and PNI, provided more persistent prognostic information for OS than short-term PFS. Exploratory age-stratified analyses and Ridge-based threshold analyses further suggest that this interpretable framework may help refine follow-up and prioritize earlier nutritional assessment or supportive care in higher-risk elderly subgroups, while external validation remains necessary before clinical implementation.

## Introduction

Esophageal squamous cell carcinoma (ESCC) is among the most common gastrointestinal malignancies worldwide and remains the predominant histological subtype of esophageal cancer in China. Its incidence rises with age, and ongoing population ageing has increased the proportion of newly diagnosed elderly patients, thereby intensifying the challenge of balancing tumor control, treatment tolerance, and supportive care (1, 2). For elderly patients with locally advanced ESCC who are not candidates for surgery, definitive chemoradiotherapy remains a cornerstone curative treatment (3). Even so, clinical outcomes remain heterogeneous, and prognostic assessment based solely on anatomical staging is often insufficient for individualized decision-making. Although the TNM system remains indispensable, it does not fully capture host-related features such as nutritional reserve, systemic inflammatory status, and treatment tolerance, which may be particularly relevant in elderly adults (4, 5).

Accumulating evidence indicates that systemic inflammatory response and nutritional status are important host-related determinants of disease progression, treatment tolerance, and long-term prognosis in malignant tumors (6). These factors are also biologically linked to the host immune milieu, because malnutrition, chronic inflammation, and impaired physiologic reserve may jointly shape immune competence and the capacity to tolerate anticancer therapy. In elderly patients with ESCC, tumor-related dysphagia, cancer-associated metabolic disturbance, and treatment-related toxicity often interact to reinforce a cycle of malnutrition and chronic inflammation. Composite nutritional-inflammatory biomarkers derived from routine blood tests have therefore emerged as pragmatic clinical surrogates of host immune-inflammatory condition in ESCC (7). Among these, the geriatric nutritional risk index (GNRI) and prognostic nutritional index (PNI) are widely used in clinical practice, and several studies have supported their prognostic relevance in ESCC (8, 9). In parallel, inflammation-related biomarkers such as the systemic inflammation response index (SIRI) and neutrophil-to-lymphocyte ratio (NLR) have also been associated with systemic inflammatory status and tumor aggressiveness (10).

Most previous studies have focused on the prognostic value of individual biomarkers and may therefore inadequately reflect the interplay among tumor burden, treatment exposure, and host condition. In this context, machine learning and related data-driven approaches offer a practical way to integrate multidimensional variables and accommodate potential nonlinear associations. In ESCC, several studies have developed prognostic models using transcriptomic, radiomic, or other multimodal features and have reported encouraging predictive performance (11–15). However, these approaches may be resource-intensive and are not always readily transferable to routine clinical practice. Interpretable models based on routinely available variables therefore remain needed, particularly for elderly patients with locally advanced ESCC receiving definitive radiotherapy. In addition, few studies have systematically compared conventional survival models, regularized regression models, and ensemble learning approaches in this setting, and interpretability analyses remain limited, thereby constraining both clinical applicability and the translational understanding of how routine nutritional-inflammatory surrogates relate to outcome heterogeneity (16, 17).

We therefore retrospectively assembled a complete-case cohort of 308 elderly patients with locally advanced ESCC treated with definitive radiotherapy and integrated clinicopathological characteristics with routinely available nutritional-inflammatory biomarkers. By comparing nine survival models for OS and PFS and applying SHAP-based interpretation to the better-performing approaches, we aimed to evaluate whether an interpretable framework based on routinely available variables could support management-relevant risk stratification in this setting, particularly for follow-up planning, supportive care prioritization, and multidisciplinary discussion in elderly patients. We also sought to clarify how host-related nutritional-inflammatory features, viewed as clinically accessible surrogates of immune-inflammatory vulnerability, contribute to outcome prediction in this setting.

## Materials and methods

### Study design and patient selection

This retrospective single-center study enrolled elderly patients with locally advanced ESCC who received definitive radiotherapy at the First Affiliated Hospital of Anhui Medical University between January 2016 and January 2023. Eligible patients met the following criteria: histopathologically confirmed ESCC; age 65 years or older; unresectable disease or refusal of surgery, corresponding to clinical stage cT1b–4bN0M0 or cT1–4bN+M0; definitive radiotherapy as the initial antitumor treatment without prior therapy; complete pretreatment peripheral blood test results; and available follow-up data, including survival status, disease progression, and treatment-related adverse events. Exclusion criteria were another malignant tumor, severe dysfunction of major organs likely to substantially affect prognostic assessment, Karnofsky Performance Status <70, immune or hematologic disorders, and missing data. After exclusion of 12 patients with incomplete records, 308 patients with complete-case data and stage II–IVA disease were included in the final analysis. Tumor staging was defined according to the 8th edition of the TNM classification.

This study was approved by the Ethics Committee of the First Affiliated Hospital of Anhui Medical University (Anhui, China; Approval No. PJ2025-12-39). The requirement for informed consent was waived by the institutional review board because of the retrospective design and complete anonymization of patient data.

### Treatment and follow-up

All patients received radiotherapy-based definitive treatment. According to individual clinical conditions, intensity-modulated radiotherapy (IMRT) or three-dimensional conformal radiotherapy (3D-CRT) was delivered either alone or in combination with chemotherapy. The prescribed radiation dose ranged from 50 to 68 Gy, administered in daily fractions of 1.8–2.0 Gy, 5 days per week. Target volume delineation and organ-at-risk constraints followed guideline-based clinical practice. Seventy-three percent of patients also received chemotherapy. Common regimens included 5-fluorouracil plus cisplatin or taxane-based combinations with cisplatin, nedaplatin, carboplatin, or lobaplatin. During treatment, patients were evaluated weekly for treatment tolerance and treatment-related adverse events. After treatment completion, follow-up was generally scheduled every 3 months during the first 2 years and every 6 months thereafter. Assessments included physical examination, chest computed tomography, barium swallow, 18F-fluorodeoxyglucose positron emission tomography, and tumor markers when clinically indicated.

### Data collection and definition of nutritional-inflammatory biomarkers

Baseline demographic, clinicopathological, treatment-related, and laboratory data were retrieved from the medical records. Variables included sex, age, tumor location, tumor length, T stage, N stage, TNM stage, smoking history, alcohol consumption, comorbidity status, radiation dose, and number of chemotherapy cycles. Pretreatment anthropometric measurements included body weight and height. Nutritional-inflammatory biomarkers were calculated from routinely available pretreatment blood parameters and anthropometric data. These included BMI, GNRI, PNI, NLR, platelet-to-lymphocyte ratio (PLR), systemic inflammation response index (SIRI), neutrophil-to-albumin ratio (NAR), and platelet-to-albumin ratio (PAR). The formulas were as follows: BMI = weight (kg)/height (m)^2; GNRI = 1.489 x serum albumin (g/L) + 41.7 x (current body weight/ideal body weight), with the weight ratio set to 1 when current body weight exceeded ideal body weight; PNI = serum albumin (g/L) + 5 x peripheral blood lymphocyte count (10^9/L); NLR = neutrophil count/lymphocyte count; PLR = platelet count/lymphocyte count; SIRI = neutrophil count x monocyte count/lymphocyte count; NAR = neutrophil count/serum albumin; and PAR = platelet count/serum albumin. All biomarkers were derived from pretreatment measurements obtained before initiation of radiotherapy.

### Study endpoints

The primary endpoints were OS and PFS. OS was defined as the interval from initiation of radiotherapy to death from any cause or last follow-up. PFS was defined as the interval from initiation of radiotherapy to documented disease progression, death from any cause, or last follow-up, whichever occurred first. The final follow-up date for the present analysis was January 2024.

### Model development and validation

Patients were randomly divided into training and testing cohorts at a ratio of 7:3. The training cohort was used for model development and the testing cohort for internal validation. Nine survival models were constructed for both OS and PFS: the conventional Cox proportional hazards model, LASSO-Cox regression, Ridge regression, Elastic Net regression, random survival forest (RSF), gradient boosting machine (GBM), ExtraTrees, XGBoost, and XGB_AFT. The selected algorithms were chosen to provide methodological diversity across conventional semi-parametric, penalized linear, and ensemble/boosting-based survival approaches, allowing comparison of interpretable and more flexible modeling strategies under the same feature set. Inclusion of ExtraTrees and XGB_AFT was exploratory and intended to assess whether added flexibility offered incremental value in this elderly radiotherapy cohort. All candidate clinicopathological and nutritional-inflammatory variables were entered using their original recorded scales. No additional manual standardization was applied before model fitting; algorithm-specific internal scaling was allowed where implemented by the respective package. Hyperparameters were optimized in the training cohort according to the characteristics of each algorithm. For regularized Cox models implemented with the glmnet package, the penalty parameter (lambda) was selected by 10-fold cross-validation using partial likelihood deviance. For tree-based ensemble methods, RSF (randomForestSRC) and ExtraTrees (ranger) were fitted with empirically conservative settings, including 600–800 trees and a minimum node size of 10–15, to reduce overfitting in the present sample. Boosting-based models were developed using gbm and xgboost. XGBoost models with Cox and AFT objectives were trained with a learning rate of 0.03, a maximum tree depth of 3, and 1200 boosting iterations. Model fitting and validation were performed using the same predefined feature set to ensure comparability across methods. These settings were intentionally conservative given the modest sample size and correlated feature structure. The relatively poor performance of some complex models was interpreted as a likely consequence of limited sample size, censoring structure, modest signal-to-noise ratio, and susceptibility to overfitting or unstable estimation despite tuning.

### Model evaluation and interpretation

The concordance index (C-index) was used to assess overall discrimination in patient risk ranking. Time-dependent AUCs were calculated to characterize predictive performance across follow-up. In line with the natural history of progression and survival, evaluation time points were set at 24, 36, and 48 months for OS and at 12, 24, and 36 months for PFS. The integrated Brier score (IBS) was additionally used to summarize prediction error over follow-up. To assess performance stability, 95% confidence intervals were estimated by bootstrap resampling (n=300). SHAP analysis was applied to the better-performing models to quantify variable contributions and to examine temporal changes in feature importance across prediction horizons. Model interpretation was considered alongside discrimination, calibration, and decision-curve findings throughout the comparative evaluation. Exploratory subgroup analyses were additionally performed according to age group (65–74 vs ≥75 years) to assess whether model performance differed within the elderly cohort. To improve clinical interpretability, threshold-based risk stratification was further conducted for the selected full-cohort models, and optimal predicted-risk cutoffs were used to generate binary and tertile risk groups in the independent testing cohort.

### Statistical analysis

Continuous variables were summarized as means with standard deviations or medians with interquartile ranges, as appropriate, and categorical variables were presented as frequencies and percentages. Baseline characteristics were compared between the training and testing cohorts using standard statistical tests according to variable type and distribution. Complete-case analysis was used for all descriptive, modeling, and validation procedures. All statistical analyses and model development procedures were performed in R software (version 4.5.1). A two-sided P value < 0.05 was considered statistically significant where applicable.

## Results

### Patient characteristics

A total of 308 elderly patients with locally advanced ESCC treated with definitive radiotherapy were included after exclusion of 12 patients with incomplete data. The cohort was randomly divided into a training set of 214 patients and a testing set of 94 patients. Median OS and PFS for the overall cohort were 19 and 13 months, respectively. Baseline demographic, clinicopathological, treatment-related, and nutritional-inflammatory characteristics were generally well balanced between the two cohorts, with no significant between-group differences in major variables (all P > 0.05). Key nutritional-inflammatory biomarkers, including GNRI, PAR, NAR, PNI, SIRI, NLR, and PLR, were similarly distributed between the training and testing cohorts, supporting the suitability of the split-sample design for internal validation. Baseline characteristics are summarized in **Table 1**.

**Table 1:** Baseline clinicopathological, treatment-related, and nutritional-inflammatory characteristics of the training and testing cohorts.

Variable	Level	Overall	Test	Train	P
Number		308	94	214	
Sex (%)	Female	105 (34.1)	28 (29.8)	77 (36.0)	0.355
	Male	203 (65.9)	66 (70.2)	137 (64.0)	
Age (median [IQR])		74.00 [70.00, 79.00]	74.00 [69.25, 79.00]	74.00 [70.00, 79.00]	0.872
Comorbidity (%)	No	196 (63.6)	65 (69.1)	131 (61.2)	0.228
	Yes	112 (36.4)	29 (30.9)	83 (38.8)	
Smoking (%)	No	254 (82.5)	84 (89.4)	170 (79.4)	0.052
	Yes	54 (17.5)	10 (10.6)	44 (20.6)	
Drinking (%)	No	252 (81.8)	78 (83.0)	174 (81.3)	0.85
	Yes	56 (18.2)	16 (17.0)	40 (18.7)	
Tumor location (%)	Cervical/upper	88 (28.6)	31 (33.0)	57 (26.6)	0.318
	Middle/lower	220 (71.4)	63 (67.0)	157 (73.4)	
Tumor length (median [IQR])		5.00 [3.38, 7.00]	5.00 [4.00, 6.00]	5.00 [3.00, 7.00]	0.796
Radiation dose (Gy) (median [IQR])		60.00 [60.00, 62.00]	60.00 [60.00, 62.00]	60.00 [60.00, 62.00]	0.95
Chemotherapy cycles (%)	≤2	198 (64.3)	56 (59.6)	142 (66.4)	0.31
	≥3	110 (35.7)	38 (40.4)	72 (33.6)	
T stage (%)	2	71 (23.1)	19 (20.2)	52 (24.3)	0.711
	3	195 (63.3)	61 (64.9)	134 (62.6)	
	4	42 (13.6)	14 (14.9)	28 (13.1)	
N stage (%)	0	123 (39.9)	36 (38.3)	87 (40.7)	0.682
	1	130 (42.2)	43 (45.7)	87 (40.7)	
	2	55 (17.9)	15 (16.0)	40 (18.7)	
TNM stage (%)	II	112 (36.4)	33 (35.1)	79 (36.9)	0.898
	III	154 (50.0)	47 (50.0)	107 (50.0)	
	IVA	42 (13.6)	14 (14.9)	28 (13.1)	
BMI (median [IQR])		20.80 [19.01, 23.15]	20.74 [19.17, 24.13]	20.82 [18.79, 22.96]	0.353
GNRI (median [IQR])		100.22 [94.59, 105.13]	99.85 [93.75, 104.83]	100.50 [94.99, 105.29]	0.472
PAR (median [IQR])		4.74 [3.65, 5.83]	4.85 [3.96, 5.73]	4.67 [3.42, 5.84]	0.168
NAR (median [IQR])		0.09 [0.07, 0.12]	0.09 [0.07, 0.12]	0.09 [0.07, 0.11]	0.169
PNI (mean (SD))		48.54 (5.23)	48.57 (5.51)	48.53 (5.11)	0.951
SIRI (median [IQR])		1.03 [0.63, 1.57]	1.11 [0.61, 1.69]	1.01 [0.64, 1.48]	0.625
NLR (median [IQR])		2.68 [1.87, 3.78]	2.63 [1.85, 3.72]	2.72 [1.88, 3.82]	0.767
PLR (median [IQR])		131.11 [98.66, 193.17]	125.87 [94.57, 188.06]	131.88 [99.58, 193.59]	0.56

TNM, tumor-node-metastasis; BMI, body mass index; GNRI, geriatric nutritional risk index; PAR, platelet-to-albumin ratio; NAR, neutrophil-to-albumin ratio; PNI, prognostic nutritional index; SIRI, systemic inflammation response index; NLR, neutrophil-to-lymphocyte ratio; PLR, platelet-to-lymphocyte ratio.

### Comparative performance of survival models for OS

The comparative performance of all models in the independent testing cohort is summarized in **Supplementary Table 1**. For OS prediction, regularized Cox regression models showed slightly better discrimination than the conventional Cox model and several tree-based ensemble approaches. Ridge regression achieved the highest C-index at 0.730 (95% CI, 0.676–0.785), whereas the conventional Cox model yielded a C-index of 0.726 (95% CI, 0.672–0.784). Elastic Net and LASSO regression also performed competitively, suggesting that regularization may improve robustness when correlated clinical and biomarker features are modeled together. By contrast, the XGB_AFT model showed the weakest performance, with a C-index of 0.527 (95% CI, 0.500–0.558). Overall, the OS results favored regularized linear survival modeling in the full testing cohort, and this pattern is clinically notable because none of the more complex ensemble or boosting approaches showed a reproducible superiority across evaluation dimensions. This finding suggests that, in elderly ESCC patients treated with definitive radiotherapy, model stability and generalizability may benefit more from regularization than from added algorithmic complexity.

### Time-dependent ROC analysis for OS

**Figure 1:**
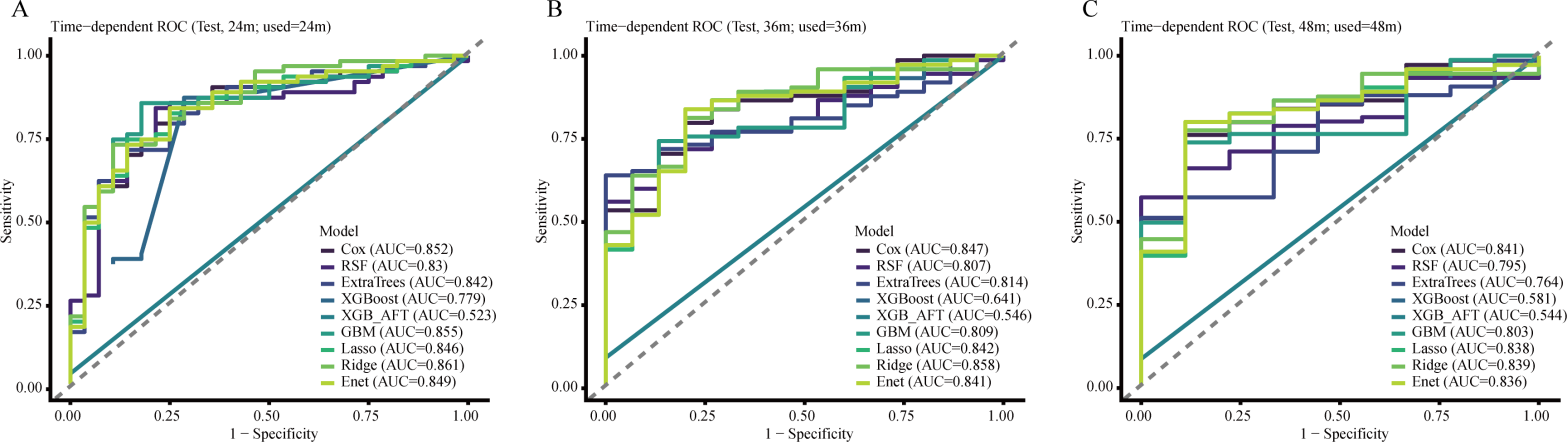
Time-dependent ROC curves for OS prediction in the independent testing cohort at 24 months **(A)**, 36 months **(B)**, and 48 months **(C)**. The diagonal dashed line indicates no discriminative ability (AUC = 0.5). Curves represent the discrimination of the statistical and machine learning models evaluated in this study. AUC, area under the curve; ROC, receiver operating characteristic; OS, overall survival.

Time-dependent ROC analysis further supported the temporal stability of the Ridge model for OS prediction. The Ridge model achieved AUCs of 0.861 at 24 months, 0.858 at 36 months, and 0.839 at 48 months. Although discrimination declined modestly over longer prediction horizons, performance remained favorable throughout follow-up. Considered together with the IBS profile, these findings indicate that Ridge regression provided the most consistent balance of discrimination and stability for OS in this internally validated cohort. Time-dependent ROC curves for OS are presented in **Figure 1**.

### Calibration and decision curve analysis for OS

**Figure 2:**
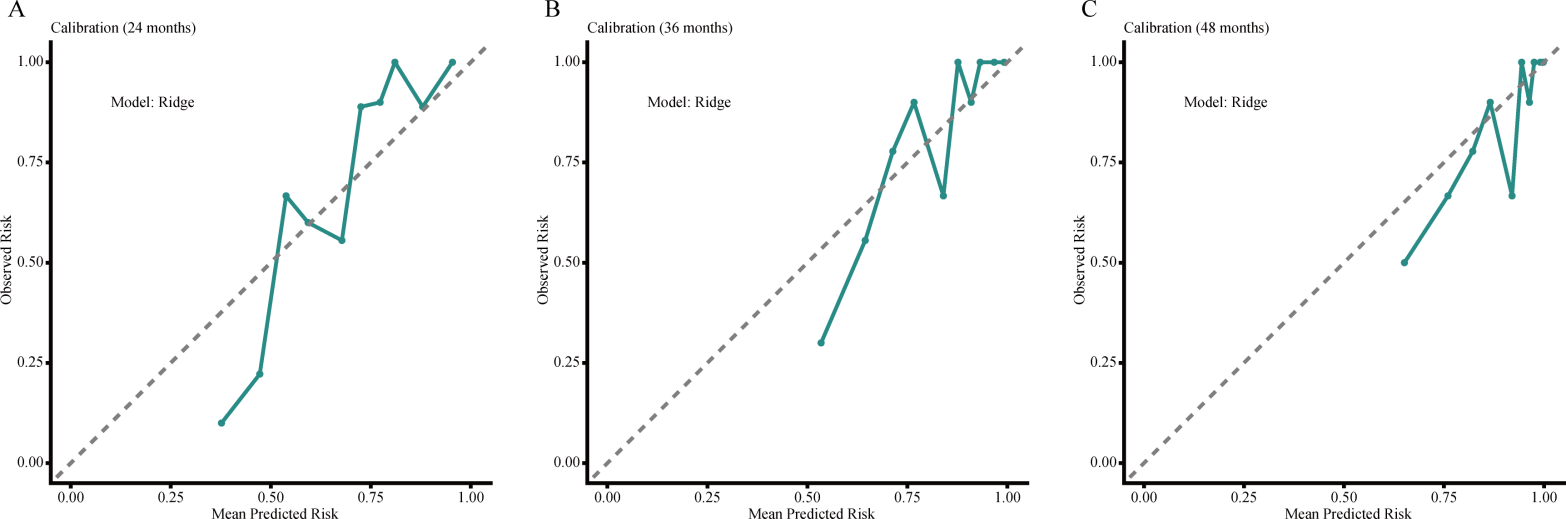
Calibration plots of the Ridge model for OS prediction at 24 months **(A)**, 36 months **(B)**, and 48 months **(C)**. The dashed diagonal line represents perfect agreement between predicted and observed risks.

**Figure 3:**
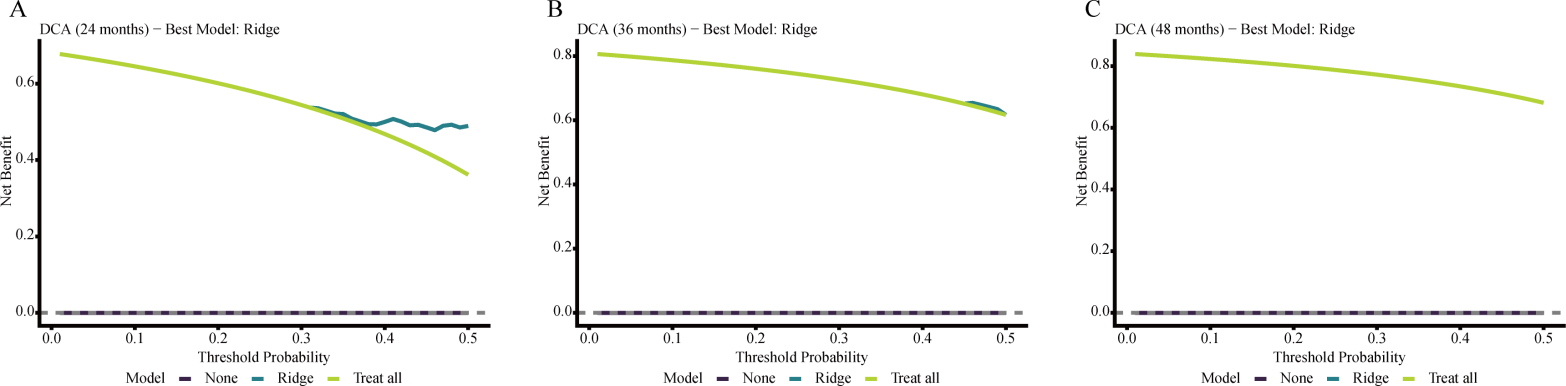
Decision curve analysis of the Ridge model for OS prediction at 24 months **(A)**, 36 months **(B)**, and 48 months **(C)**. Net benefit was compared with the default strategies of treating all patients or treating none across a range of threshold probabilities.

Further assessment of the Ridge model showed acceptable agreement between predicted and observed OS risks at 24, 36, and 48 months, although departures were evident in portions of the intermediate- and high-risk ranges, particularly at later time points. In decision curve analysis, the Ridge model provided greater net benefit than the treat-all and treat-none strategies across selected threshold probabilities, with the clearest advantage at 24 months and a smaller gain at 36 months. By 48 months, the net-benefit curve largely overlapped with the treat-all strategy within the displayed threshold range. These findings suggest that the practical value of the model was more apparent for short- to intermediate-term OS stratification, where it may be more helpful for identifying elderly patients who warrant closer surveillance, earlier supportive intervention, or more intensive multidisciplinary review, than for longer-term decision support. Calibration plots and decision curves for OS are presented in **Figures 2** and **3**.

### Comparative performance of survival models for PFS

For PFS prediction, the relative ranking of models partly resembled that observed for OS, although no single model was uniformly superior across all evaluation dimensions. ExtraTrees achieved the highest overall C-index in the testing cohort at 0.687 (95% CI, 0.634–0.739). Ridge regression and RSF followed closely, with C-indices of 0.685 (95% CI, 0.628–0.736) and 0.686 (95% CI, 0.624–0.738), respectively. XGBoost again showed the weakest discrimination, with a C-index of 0.504. For PFS, the relative ranking of models was more unstable across metrics and time horizons than for OS. Although ExtraTrees achieved the highest overall C-index in the full testing cohort, this advantage was not sustained uniformly across landmark analyses, and no complex machine-learning model showed a stable overall edge. These findings suggest that short-term progression risk in this elderly cohort may be harder to capture with a single dominant algorithm and that endpoint-specific model selection remains more appropriate than assuming superiority of more flexible methods.

### Time-dependent ROC analysis for PFS

**Figure 4:**
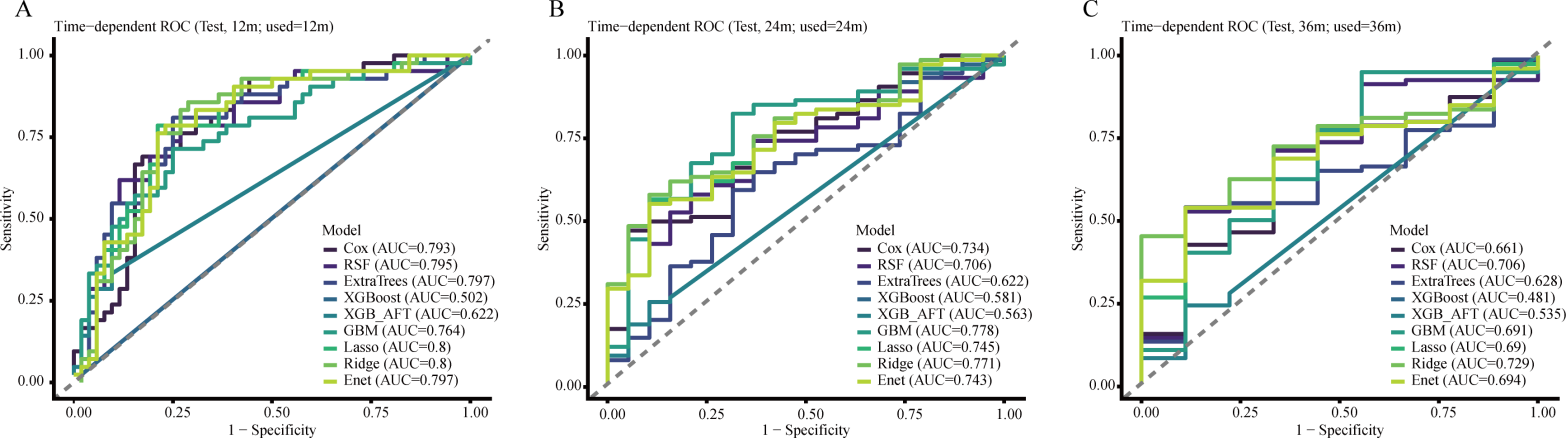
Time-dependent ROC curves for PFS prediction in the independent testing cohort at 12 months **(A)**, 24 months **(B)**, and 36 months **(C)**. The diagonal dashed line indicates no discriminative ability (AUC = 0.5). Curves represent the discrimination of the statistical and machine learning models evaluated in this study. AUC, area under the curve; ROC, receiver operating characteristic; PFS, progression-free survival.

At the prespecified landmarks, Ridge regression showed the most consistent temporal discrimination for PFS, with AUCs of 0.800, 0.771, and 0.729 at 12, 24, and 36 months, respectively. Although ExtraTrees achieved the highest overall C-index for PFS, that advantage was not maintained consistently at later follow-up landmarks, where Ridge regression showed more stable time-specific performance. Taken together, the PFS results were both endpoint- and time-dependent: ExtraTrees provided the strongest overall risk ranking, whereas Ridge offered greater medium- and longer-term stability. Time-dependent ROC curves for PFS are presented in **Figure 4**.

### Calibration and decision curve analysis for PFS

**Figure 5:**
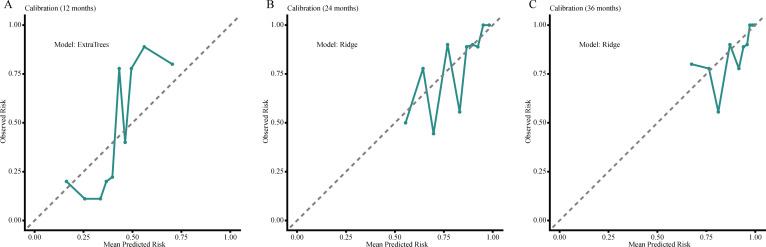
Calibration plots of the best-performing PFS models at 12 months [**(A)**; ExtraTrees model], 24 months [**(B)**; ridge model], and 36 months [**(C)**; ridge model]. The dashed diagonal line represents perfect agreement between predicted and observed risks.

**Figure 6:**
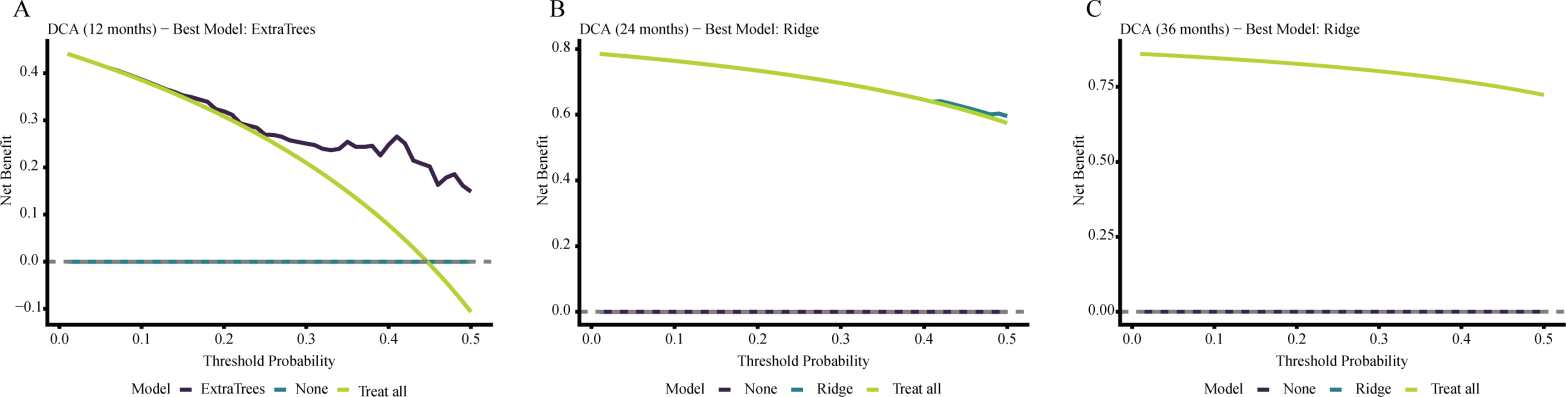
Decision curve analysis of the best-performing PFS models at 12 months [**(A)**; ExtraTrees model], 24 months [**(B)**; ridge model], and 36 months [**(C)**; ridge model]. Net benefit was compared with the default strategies of treating all patients or treating none across a range of threshold probabilities.

Calibration plots showed acceptable agreement between predicted and observed PFS risks for the selected models at 12, 24, and 36 months, namely ExtraTrees at 12 months and Ridge at 24 and 36 months. Decision curve analysis indicated net-benefit advantages over the treat-all and treat-none strategies across selected threshold probabilities, with clearer separation at earlier time points and smaller gains by 36 months. Overall, the practical value of PFS prediction appeared more variable across follow-up landmarks than that of OS prediction, suggesting that its role in management decisions may be more limited to selected clinical contexts and time points rather than broad uniform application. Calibration plots and decision curves for PFS are presented in **Figures 5** and **6**.

### SHAP-based interpretation of OS prediction

**Figure 7:**
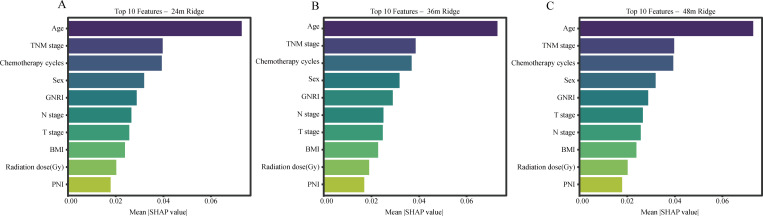
SHAP summary plots showing the top 10 predictors for OS at 24 months **(A)**, 36 months **(B)**, and 48 months **(C)** in the ridge model. Features are ordered by mean absolute SHAP value, reflecting their overall contribution to model output. Persistent high-ranking features indicate relatively stable prognostic relevance across time horizons. SHAP, Shapley Additive Explanations; OS, overall survival.

To improve interpretability, SHAP analysis was performed for the selected OS model. Across the 24-, 36-, and 48-month horizons, age, TNM stage, and number of chemotherapy cycles consistently ranked among the most influential predictors. This pattern indicates that host reserve, tumor burden, and treatment delivery jointly shaped long-term survival risk in this elderly population. Nutritional indicators retained persistent prognostic relevance for OS. GNRI, PNI, and BMI remained among the 10 most influential predictors at all evaluated time points, indicating that baseline nutritional status provided stable prognostic information. By contrast, baseline inflammatory markers such as SIRI and NLR ranked relatively low in the SHAP analyses. This should not be interpreted as evidence against the biological relevance of inflammation, but rather as indicating that, within a multivariable prediction framework integrating tumor burden, age, treatment-related variables, and host nutritional status, single baseline inflammatory indices provided less incremental prognostic information than expected. Temporal changes in feature importance for OS are shown in **Figure 7**.

### SHAP-based interpretation of PFS prediction

**Figure 8:**
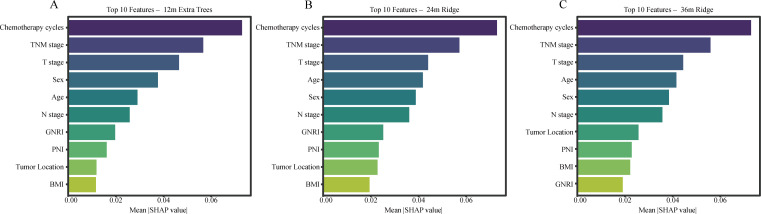
SHAP-based feature importance rankings for PFS at 12 months [**(A)**; ExtraTrees model], 24 months [**(B)**; ridge model], and 36 months [**(C)**; ridge model]. Bars represent mean absolute SHAP values, with longer bars indicating greater contribution to risk prediction. SHAP, Shapley Additive Explanations; PFS, progression-free survival.

SHAP analysis was further performed to characterize feature importance for PFS at different time horizons. Number of chemotherapy cycles, TNM stage, and T stage consistently emerged as the dominant predictors, underscoring the importance of treatment delivery and tumor extent in progression risk. Compared with OS, nutritional indicators showed less persistent importance in PFS prediction, particularly at the earlier time horizon, suggesting that short-term progression may be driven more strongly by tumor extent and treatment-related factors than by baseline nutritional reserve alone. This contrast between OS and PFS further supports the interpretation that nutritional vulnerability may exert a more sustained effect on long-term survival than on early progression events in elderly patients undergoing definitive radiotherapy. SHAP-based feature importance rankings for PFS at different time points are shown in **Figure 8**.

### Exploratory age-stratified model performance

Exploratory age-stratified analyses revealed heterogeneity in model behavior between patients aged 65–74 years and those aged ≥75 years. The highest mean C-index was defined as the average of landmark-specific C-index values within each age stratum for the corresponding endpoint. For OS, the highest mean C-index was observed with ExtraTrees in the 65–74-year subgroup and with GBM in the ≥75-year subgroup. For PFS, ExtraTrees showed the highest mean C-index in patients aged 65–74 years, whereas LASSO performed best in those aged ≥75 years. These findings suggest that prognostic structure may differ even within elderly ESCC populations and that model preference may vary by age stratum rather than remaining uniform across all older patients. Detailed age-stratified model performance is provided in **Supplementary Table 2**.

### Risk-threshold stratification and prognostic separation

**Figure 9:**
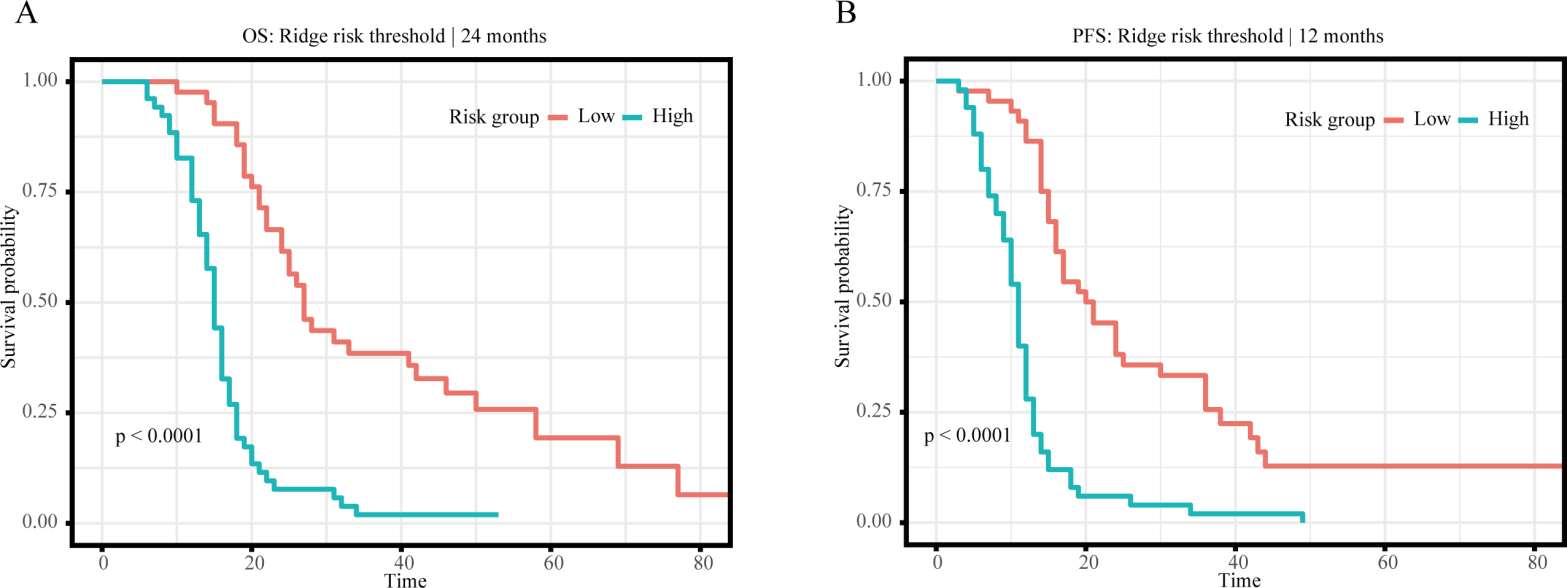
Dual-panel Kaplan–Meier curves for model-derived binary risk groups in the independent testing cohort. **(A)** Overall survival according to the 24-month Ridge-derived cutoff (predicted risk cutoff = 0.6784). **(B)** Progression-free survival according to the 12-month Ridge-derived cutoff (predicted risk cutoff = 0.3943). High-risk groups showed significantly worse outcomes than low-risk groups in both analyses.

To improve clinical interpretability, threshold-based risk stratification was performed using Ridge-derived predicted risk, because Ridge showed comparatively stable landmark discrimination and offered an interpretable, consistent framework for exploratory risk grouping across endpoints. For OS, the 24-month Ridge model identified an optimal predicted-risk cutoff of 0.6784, which separated the testing cohort into high- and low-risk groups with a hazard ratio of 4.57 (95% CI, 2.81–7.43; P < 0.001). For PFS, the 12-month Ridge model identified an optimal cutoff of 0.3943, with a corresponding hazard ratio of 3.89 (95% CI, 2.47–6.14; P < 0.001). These findings indicate that model-derived risk estimates can be translated into clinically interpretable strata rather than remaining purely statistical outputs. In the independent testing cohort, binary and tertile risk assignments also showed progressive enrichment of adverse outcomes across model-derived strata, supporting the discriminatory value of threshold-based grouping for both OS and PFS. Detailed threshold outputs and risk-group assignments are provided in **Supplementary Table 3**. Kaplan–Meier analysis showed clear survival separation between the model-derived high- and low-risk groups for both OS and PFS (**Figure 9**).

## Discussion

In this study, we examined elderly patients with locally advanced ESCC treated with definitive radiotherapy, integrated clinicopathological characteristics with nutritional-inflammatory biomarkers, and compared the prognostic performance of conventional survival models, regularized Cox regression models, and ensemble learning approaches. Several findings merit emphasis. Ridge regression showed the most consistent performance for OS, whereas no single model dominated PFS across all evaluation dimensions. The observation that age, TNM stage, and treatment delivery ranked highly is broadly in line with established clinical knowledge. A key novelty of the present study is not simply that nutritional-inflammatory variables were included, but that their value was examined within a comparative, interpretable survival-modeling framework tailored to an elderly definitive-radiotherapy cohort. In this setting, more complex machine-learning methods did not demonstrate a stable performance advantage, whereas regularized linear models showed greater robustness for OS. This is clinically relevant because it argues against equating algorithmic complexity with better prognostic utility in older, moderately sized real-world datasets. Taken together, these findings support the potential utility of an interpretable risk-stratification framework in elderly ESCC, while also underscoring the relevance of routine nutritional-inflammatory variables as clinically accessible indicators of host immune-inflammatory vulnerability in this population, albeit requiring cautious interpretation and external validation.

With respect to model performance, our findings indicate that regularized Cox regression was particularly well suited to OS prediction in this dataset. Although Ridge regression achieved the highest C-index, its numerical advantage over the conventional Cox model was modest. In real-world datasets characterized by limited sample size, moderate feature dimensionality, and correlated predictors, the additional flexibility of highly complex nonlinear algorithms does not necessarily translate into better generalization (12, 18). By contrast, ridge-penalized regression applies L2 regularization to shrink unstable coefficients while retaining all variables, which may improve robustness when clinicopathological and nutritional-inflammatory features are interrelated (19). The relatively poor performance of some boosting-based approaches in the present cohort is therefore likely to reflect sample-size constraints, censoring structure, and limited signal-to-noise ratio rather than an intrinsic lack of clinical relevance of those methods. From a clinical perspective, this is important because prognostic tools intended for routine practice must balance accuracy with transparency and stability. The PFS findings further reinforce this point. Although ExtraTrees showed the highest overall discrimination, that advantage was small and was not maintained consistently at later landmarks, whereas Ridge delivered more stable time-specific performance. Rather than suggesting that one universally optimal algorithm exists, these results favor endpoint-specific model selection over the assumption that greater algorithmic complexity will necessarily yield better clinical prediction (17).

Beyond discrimination, calibration and decision-curve analyses offered a more clinically informative view of model performance. For OS, the Ridge model showed acceptable calibration and a net-benefit advantage within selected threshold ranges, most clearly at 24 months, although this benefit became less distinct at later time points. For PFS, the selected models also showed acceptable calibration, but the extent and consistency of net benefit varied across follow-up landmarks. The threshold analyses further strengthen the translational relevance of the present framework. Within this framework, patients with a model-predicted 24-month OS risk >0.6784 or a model-predicted 12-month PFS risk >0.3943 may be considered high-risk. In clinical practice, these risk thresholds may help identify elderly patients who warrant earlier nutritional assessment, closer monitoring of treatment tolerance, shorter follow-up intervals, or earlier multidisciplinary review.

According to the SHAP analysis, age, TNM stage, and number of chemotherapy cycles ranked prominently in both OS and PFS prediction. This result is not intended to suggest novel biological determinants; rather, it confirms that the modeling framework appropriately captured clinically recognized sources of prognostic heterogeneity in elderly patients with locally advanced ESCC treated with definitive radiotherapy or chemoradiotherapy (20). Elderly patients often have reduced physiological reserve, lower tolerance to chemoradiotherapy, and a higher risk of treatment-related adverse events, all of which may influence long-term survival (2). The TNM staging system remains central to prognostic assessment because it captures the anatomical extent of disease, including tumor invasion depth, nodal involvement, and distant metastasis, all of which are closely linked to survival risk (21). The contribution of chemotherapy cycle number warrants careful interpretation. Rather than representing a directly causal baseline characteristic, it more plausibly functions as a marker of treatment delivery, treatment tolerance, physician selection, and overall clinical fitness. Accordingly, the present framework is better understood as a treatment-integrated prognostic model than as a strictly pretreatment tool. At the same time, prior evidence suggests that, in elderly patients with locally advanced ESCC receiving definitive radiotherapy, chemoradiotherapy is associated with improved OS and PFS compared with radiotherapy alone (22).

Our findings also support the prognostic relevance of nutritional biomarkers in elderly patients with ESCC receiving definitive radiotherapy. GNRI, PNI, and BMI consistently remained among the 10 most influential predictors for OS, and their relative importance was stable across follow-up horizons. This observation extends previous evidence linking nutritional vulnerability to survival in ESCC to an elderly population with locally advanced disease treated with definitive radiotherapy (23, 24). In elderly patients, malnutrition may also reflect clinician selection for chemotherapy and treatment tolerance, increase the risk of adverse outcomes, and weaken host antitumor immunity (25, 26). By contrast, the contribution of nutritional indicators to PFS was less stable over time, suggesting that progression may be shaped more strongly by tumor-related and treatment-related factors, whereas nutritional status may exert a more sustained influence on long-term survival.

Recent evidence further supports the clinical relevance of nutritional support in esophageal cancer treated with chemoradiotherapy. A previous study showed that interdisciplinary nutritional support during concurrent chemoradiotherapy helped maintain nutritional status, improve treatment compliance, and reduce hospitalization burden in patients with esophageal cancer (27). In addition, a review focused specifically on older patients undergoing chemoradiotherapy emphasized that dysphagia, comorbidity burden, age-related physiologic changes, and muscle loss contribute to malnutrition risk, and that early nutritional screening and proactive intervention may help improve treatment tolerance and facilitate treatment completion in this population (28). Combined with our SHAP findings, these data support the view that host-condition markers in ESCC may reflect a clinically relevant interplay between nutritional reserve, systemic inflammatory burden, immune status, and tolerance to definitive therapy. This may be particularly important in elderly patients, in whom higher-risk profiles based on routine nutritional variables could help identify those who may benefit from earlier structured nutritional assessment, closer monitoring of treatment tolerance, and more proactive supportive care throughout radiotherapy and systemic treatment (29, 30).

The fact that inflammatory biomarkers did not rank among the most influential features in the SHAP analysis should not be interpreted as evidence that immune-related processes are unimportant in elderly ESCC. Rather, it highlights a limitation of the present dataset and of routine single-time-point blood-based surrogates. In this study, all inflammatory markers were assessed only at baseline, whereas the host immune-inflammatory state during definitive radiotherapy is dynamic and may be shaped by tumor burden, dysphagia-related malnutrition, occult infection, tissue injury, treatment tolerance, and systemic stress responses. Accordingly, indices such as SIRI and NLR should be viewed as low-resolution clinical proxies rather than direct mechanistic readouts of antitumor immunity. Previous studies have linked both baseline SIRI and post-treatment changes in systemic inflammatory burden with survival in ESCC, suggesting that longitudinal assessment may be more informative than a single measurement (31). Importantly, the relatively weak SHAP ranking of NLR or SIRI should not be conflated with failure of inflammation-related biology; rather, it indicates that this study was designed as a multivariable prediction exercise rather than a conventional independent-factor screening analysis, and that baseline inflammatory surrogates contributed less incremental information than nutritional or tumor-burden variables once these were modeled jointly. The immunology-related relevance of the present study therefore lies not in defining specific immune pathways, but in showing that routinely available nutritional-inflammatory markers may capture part of the host immune-inflammatory vulnerability associated with survival heterogeneity in this elderly radiotherapy cohort. Accordingly, SHAP-based feature rankings should be interpreted as comparative model attributions, not as mechanistic inference about host immunity. Future studies incorporating dynamic inflammatory trajectories, immune-cell phenotyping, or other molecularly informed biomarkers may clarify this relationship more fully.

The exploratory age-stratified analyses add an additional layer of clinical nuance. Patients aged 65–74 years and those aged ≥75 years did not share the same top-ranked models for either OS or PFS, suggesting that the prognostic architecture within elderly ESCC is itself heterogeneous. This may reflect age-related differences in physiologic reserve, competing mortality risk, treatment tolerance, and the relative contribution of host-condition variables. These findings suggest that elderly patients should not be considered a fully homogeneous group when interpreting prognostic model performance in definitive radiotherapy settings.

This study has several strengths. It focused on an elderly population with locally advanced ESCC treated with definitive radiotherapy, a group that remains underrepresented in prognostic modeling research. We also compared multiple model classes across two clinically relevant endpoints and integrated discrimination, calibration, decision-curve analysis, and SHAP-based interpretation across different time horizons. In addition, we incorporated a broad set of routinely available nutritional-inflammatory biomarkers, allowing host-related features to be evaluated alongside tumor- and treatment-related variables. Although this was not a mechanistic immunology or multi-omics study, it addressed a complementary translational question: whether readily obtainable clinicopathological and blood-derived nutritional-inflammatory markers can support interpretable risk stratification in routine oncology practice while reflecting clinically relevant aspects of host immune-inflammatory condition.

Several limitations should also be acknowledged. First, this was a retrospective single-center study and therefore remains vulnerable to selection bias. Second, only complete-case data were analyzed, which improved internal consistency but may also have introduced selection effects related to data availability. Third, although bootstrap resampling and an internal test cohort were used to improve robustness, the single random train-test split may still introduce partition dependence. Accordingly, these methods should currently be regarded as investigational tools for prognostic enrichment and management-oriented stratification rather than systems ready to direct routine clinical decisions independently, pending external validation in independent multicenter cohorts and prospective workflow evaluation. Fourth, only baseline nutritional-inflammatory biomarkers were analyzed, whereas dynamic changes during and after radiotherapy may provide additional prognostic information. Finally, this study did not incorporate radiomic, transcriptomic, spatial immune, or other multi-omics features. Even so, the pragmatic feature set can also be viewed as a strength because it reflects the type of information readily obtainable in everyday oncology practice. Future work should determine whether integrating longitudinal host-related biomarkers with multimodal or multi-omics data yields clinically meaningful incremental benefit beyond interpretable models based on routine variables.

## Conclusion

In conclusion, this study systematically compared multiple survival modeling strategies in elderly patients with locally advanced ESCC treated with definitive radiotherapy and showed that model performance was endpoint dependent. Ridge regression showed the most consistent performance for OS, whereas no single model was uniformly superior for PFS across all evaluation metrics, supporting an exploratory rather than definitive interpretation of model ranking. Nutritional-inflammatory biomarkers, particularly GNRI, PNI, and BMI, contributed stable prognostic information, especially for OS. Calibration and decision-curve analyses further supported the overall performance of the selected models, and the exploratory age-stratified findings also suggest that prognostic model behavior may differ between younger-old and oldest-old patients. Ridge-based threshold analyses further showed that model-derived predicted risk could be translated into clinically meaningful high- and low-risk groups and may help prioritize earlier nutritional assessment and closer supportive monitoring in higher-risk elderly patients. External validation and future incorporation of dynamic or multi-omics biomarkers will be necessary before broader clinical application.

## Data Availability

The original contributions presented in the study are included in the article/**Supplementary Material**. Further inquiries can be directed to the corresponding authors.
