# Supplementary material for: Interpretable survival modeling integrating nutritional-inflammatory biomarkers in elderly patients with locally advanced esophageal squamous cell carcinoma treated with definitive radiotherapy
Source: Front Immunol. 2026 May 11;17:1836567. doi: 10.3389/fimmu.2026.1836567 (PMC13199357; doi:10.3389/fimmu.2026.1836567)
Supplement: Supplementary file 1 [file DataSheet1.pdf]

Supplementary Table 1 Predictive performance of statistical and machine learning models for OS and PFS in the independent testing cohort.

Model Name	Time	C-Index (95% CI)	AUC (95% CI)	IBS (95% CI)
**OS**				
Cox	24	0.726 (0.672 - 0.784)	0.852 (0.758 - 0.924)	0.144 (0.110 - 0.178)
Cox	36	0.726 (0.665 - 0.783)	0.847 (0.742 - 0.927)	0.115 (0.079 - 0.156)
Cox	48	0.726 (0.668 - 0.776)	0.841 (0.733 - 0.960)	0.103 (0.063 - 0.144)
Enet	24	0.725 (0.667 - 0.787)	0.849 (0.758 - 0.920)	0.148 (0.121 - 0.180)
Enet	36	0.725 (0.665 - 0.786)	0.841 (0.729 - 0.927)	0.113 (0.079 - 0.157)
Enet	48	0.725 (0.664 - 0.778)	0.836 (0.710 - 0.952)	0.099 (0.057 - 0.144)
ExtraTrees	24	0.704 (0.660 - 0.755)	0.842 (0.754 - 0.924)	0.169 (0.145 - 0.191)
ExtraTrees	36	0.710 (0.652 - 0.759)	0.814 (0.733 - 0.893)	0.135 (0.104 - 0.167)
ExtraTrees	48	0.683 (0.628 - 0.740)	0.764 (0.642 - 0.899)	0.118 (0.079 - 0.160)
GBM	24	0.719 (0.662 - 0.769)	0.855 (0.770 - 0.931)	0.144 (0.110 - 0.180)
GBM	36	0.719 (0.663 - 0.770)	0.809 (0.693 - 0.903)	0.123 (0.083 - 0.172)
GBM	48	0.720 (0.658 - 0.772)	0.803 (0.679 - 0.915)	0.110 (0.060 - 0.166)
Lasso	24	0.726 (0.667 - 0.789)	0.846 (0.758 - 0.919)	0.151 (0.124 - 0.182)
Lasso	36	0.726 (0.665 - 0.788)	0.842 (0.730 - 0.929)	0.113 (0.078 - 0.157)
Lasso	48	0.726 (0.661 - 0.780)	0.838 (0.707 - 0.953)	0.099 (0.057 - 0.145)
Ridge	24	0.730 (0.681 - 0.788)	0.861 (0.780 - 0.927)	0.152 (0.123 - 0.177)
Ridge	36	0.730 (0.676 - 0.785)	0.858 (0.761 - 0.940)	0.114 (0.080 - 0.153)
Ridge	48	0.730 (0.674 - 0.777)	0.839 (0.715 - 0.951)	0.102 (0.059 - 0.148)
RSF	24	0.714 (0.665 - 0.767)	0.830 (0.731 - 0.911)	0.165 (0.138 - 0.190)
RSF	36	0.711 (0.656 - 0.762)	0.807 (0.710 - 0.897)	0.126 (0.090 - 0.166)
RSF	48	0.711 (0.659 - 0.759)	0.795 (0.689 - 0.905)	0.110 (0.062 - 0.165)
XGB_AFT	24	0.527 (0.500 - 0.558)	0.523 (0.500 - 0.549)	0.683 (0.582 - 0.779)
XGB_AFT	36	0.531 (0.503 - 0.569)	0.546 (0.520 - 0.577)	0.819 (0.736 - 0.891)
XGB_AFT	48	0.531 (0.503 - 0.573)	0.544 (0.513 - 0.579)	0.858 (0.768 - 0.934)
XGBoost	24	0.657 (0.593 - 0.714)	0.779 (0.670 - 0.881)	0.462 (0.375 - 0.561)
XGBoost	36	0.597 (0.548 - 0.644)	0.641 (0.502 - 0.776)	0.182 (0.110 - 0.253)
XGBoost	48	0.552 (0.520 - 0.591)	0.581 (0.456 - 0.731)	0.142 (0.080 - 0.216)
**PFS**				
Cox	12	0.677 (0.630 - 0.728)	0.793 (0.709 - 0.876)	0.193 (0.157 - 0.228)

Model Name	Time	C-Index (95% CI)	AUC (95% CI)	IBS (95% CI)
Cox	24	0.677 (0.630 - 0.722)	0.734 (0.607 - 0.844)	0.151 (0.107 - 0.200)
Cox	36	0.677 (0.623 - 0.723)	0.661 (0.457 - 0.832)	0.124 (0.079 - 0.177)
Enet	12	0.676 (0.620 - 0.724)	0.797 (0.699 - 0.881)	0.196 (0.167 - 0.227)
Enet	24	0.676 (0.628 - 0.724)	0.743 (0.615 - 0.850)	0.144 (0.099 - 0.189)
Enet	36	0.676 (0.621 - 0.723)	0.694 (0.523 - 0.844)	0.112 (0.068 - 0.164)
ExtraTrees	12	0.687 (0.634 - 0.739)	0.797 (0.703 - 0.883)	0.201 (0.175 - 0.231)
ExtraTrees	24	0.597 (0.544 - 0.649)	0.622 (0.479 - 0.750)	0.165 (0.124 - 0.204)
ExtraTrees	36	0.579 (0.516 - 0.638)	0.628 (0.436 - 0.803)	0.118 (0.081 - 0.162)
GBM	12	0.669 (0.604 - 0.729)	0.764 (0.662 - 0.854)	0.200 (0.163 - 0.245)
GBM	24	0.669 (0.605 - 0.721)	0.778 (0.643 - 0.887)	0.146 (0.094 - 0.201)
GBM	36	0.669 (0.611 - 0.724)	0.691 (0.475 - 0.860)	0.108 (0.064 - 0.165)
Lasso	12	0.677 (0.622 - 0.723)	0.800 (0.704 - 0.881)	0.193 (0.163 - 0.225)
Lasso	24	0.677 (0.628 - 0.724)	0.745 (0.617 - 0.854)	0.145 (0.101 - 0.190)
Lasso	36	0.677 (0.620 - 0.724)	0.690 (0.515 - 0.851)	0.114 (0.070 - 0.167)
Ridge	12	0.685 (0.628 - 0.736)	0.800 (0.709 - 0.887)	0.200 (0.172 - 0.231)
Ridge	24	0.685 (0.630 - 0.728)	0.771 (0.644 - 0.872)	0.140 (0.097 - 0.184)
Ridge	36	0.685 (0.634 - 0.728)	0.729 (0.577 - 0.854)	0.109 (0.066 - 0.160)
RSF	12	0.686 (0.624 - 0.738)	0.795 (0.700 - 0.882)	0.201 (0.176 - 0.232)
RSF	24	0.625 (0.576 - 0.672)	0.706 (0.569 - 0.813)	0.154 (0.107 - 0.198)
RSF	36	0.617 (0.558 - 0.679)	0.706 (0.516 - 0.880)	0.107 (0.065 - 0.159)
XGB_AFT	12	0.558 (0.518 - 0.601)	0.622 (0.552 - 0.703)	0.447 (0.345 - 0.553)
XGB_AFT	24	0.561 (0.514 - 0.608)	0.563 (0.465 - 0.641)	0.789 (0.714 - 0.874)
XGB_AFT	36	0.575 (0.518 - 0.626)	0.535 (0.354 - 0.680)	0.870 (0.797 - 0.935)
XGBoost	12	0.504 (0.501 - 0.672)	0.502 (0.478 - 0.536)	0.457 (0.351 - 0.559)
XGBoost	24	0.561 (0.520 - 0.607)	0.581 (0.478 - 0.703)	0.240 (0.149 - 0.339)
XGBoost	36	0.514 (0.501 - 0.624)	0.481 (0.453 - 0.500)	0.151 (0.085 - 0.232)

Supplementary Table 2 Age-stratified comparative model performance for OS and PFS.

Endpoint	Age group (years)	Model name	Time (months)	C-index	AUC	IBS
OS	65-74	Cox	24	0.647	0.725	0.221
OS	65-74	Lasso	24	0.650	0.700	0.230
OS	65-74	Ridge	24	0.626	0.715	0.224
OS	65-74	Enet	24	0.531	0.685	0.234
OS	65-74	RSF	24	0.599	0.698	0.220
OS	65-74	GBM	24	0.628	0.727	0.224
OS	65-74	ExtraTrees	24	0.666	0.636	0.238
OS	65-74	XGBoost	24	NA	0.564	0.462
OS	65-74	XGB_AFT	24	NA	0.500	0.521
OS	65-74	Cox	36	0.657	0.729	0.184
OS	65-74	Lasso	36	0.650	0.683	0.192
OS	65-74	Ridge	36	0.626	0.736	0.181
OS	65-74	Enet	36	0.572	0.688	0.192
OS	65-74	RSF	36	0.614	0.729	0.180
OS	65-74	GBM	36	0.628	0.744	0.177
OS	65-74	ExtraTrees	36	0.666	0.698	0.186
OS	65-74	XGBoost	36	NA	0.636	0.250
OS	65-74	XGB_AFT	36	NA	0.500	0.717
OS	65-74	Cox	48	0.644	0.674	0.166
OS	65-74	Lasso	48	0.650	0.576	0.139
OS	65-74	Ridge	48	0.626	0.671	0.133
OS	65-74	Enet	48	0.526	0.591	0.136
OS	65-74	RSF	48	0.586	0.666	0.132
OS	65-74	GBM	48	0.628	0.757	0.141
OS	65-74	ExtraTrees	48	0.666	0.568	0.146
OS	65-74	XGBoost	48	NA	0.504	0.195
OS	65-74	XGB_AFT	48	NA	0.500	0.835
OS	≥75	Cox	24	0.686	0.843	0.134
OS	≥75	Lasso	24	0.703	0.877	0.125
OS	≥75	Ridge	24	0.689	0.831	0.132
OS	≥75	Enet	24	0.503	0.874	0.135
OS	≥75	RSF	24	0.635	0.868	0.140
OS	≥75	GBM	24	0.717	0.840	0.145
OS	≥75	ExtraTrees	24	0.627	0.796	0.145
OS	≥75	XGBoost	24	0.511	0.540	0.211
OS	≥75	XGB_AFT	24	NA	0.557	0.787
OS	≥75	Cox	36	0.669	0.883	0.087
OS	≥75	Lasso	36	0.703	0.868	0.082
OS	≥75	Ridge	36	0.689	0.856	0.083
OS	≥75	Enet	36	NA	0.853	0.086
OS	≥75	RSF	36	0.674	0.863	0.088
OS	≥75	GBM	36	0.717	0.854	0.097
OS	≥75	ExtraTrees	36	0.627	0.863	0.095
OS	≥75	XGBoost	36	0.511	0.500	0.111
OS	≥75	XGB_AFT	36	NA	0.550	0.889
OS	≥75	Cox	48	0.664	0.887	0.089
OS	≥75	Lasso	48	0.703	0.901	0.084
OS	≥75	Ridge	48	0.689	0.901	0.084
OS	≥75	Enet	48	NA	0.899	0.089
OS	≥75	RSF	48	0.663	0.934	0.089
OS	≥75	GBM	48	0.717	0.859	0.104
OS	≥75	ExtraTrees	48	0.632	0.928	0.090
OS	≥75	XGBoost	48	0.516	0.500	0.111
OS	≥75	XGB_AFT	48	NA	0.562	0.889
PFS	65-74	Cox	12	0.692	0.739	0.202

Endpoint	Age group (years)	Model name	Time (months)	C-index	AUC	IBS
PFS	65-74	Lasso	12	0.654	0.746	0.185
PFS	65-74	Ridge	12	0.657	0.742	0.185
PFS	65-74	Enet	12	NA	0.754	0.183
PFS	65-74	RSF	12	0.724	0.788	0.171
PFS	65-74	GBM	12	0.661	0.714	0.188
PFS	65-74	ExtraTrees	12	0.703	0.838	0.155
PFS	65-74	XGBoost	12	NA	0.500	0.292
PFS	65-74	XGB_AFT	12	NA	0.500	0.292
PFS	65-74	Cox	24	0.698	0.830	0.159
PFS	65-74	Lasso	24	0.654	0.772	0.172
PFS	65-74	Ridge	24	0.657	0.743	0.184
PFS	65-74	Enet	24	0.577	0.752	0.178
PFS	65-74	RSF	24	0.700	0.772	0.188
PFS	65-74	GBM	24	0.661	0.794	0.186
PFS	65-74	ExtraTrees	24	0.703	0.786	0.188
PFS	65-74	XGBoost	24	0.519	0.603	0.312
PFS	65-74	XGB_AFT	24	NA	0.531	0.688
PFS	65-74	Cox	36	0.696	0.742	0.161
PFS	65-74	Lasso	36	0.654	0.640	0.162
PFS	65-74	Ridge	36	0.657	0.661	0.145
PFS	65-74	Enet	36	0.567	0.621	0.154
PFS	65-74	RSF	36	0.658	0.818	0.132
PFS	65-74	GBM	36	0.661	0.793	0.129
PFS	65-74	ExtraTrees	36	0.703	0.747	0.142
PFS	65-74	XGBoost	36	0.525	0.678	0.145
PFS	65-74	XGB_AFT	36	NA	0.576	0.819
PFS	≥75	Cox	12	0.704	0.842	0.168
PFS	≥75	Lasso	12	0.720	0.802	0.180
PFS	≥75	Ridge	12	0.711	0.817	0.179
PFS	≥75	Enet	12	NA	0.810	0.180
PFS	≥75	RSF	12	0.669	0.834	0.205
PFS	≥75	GBM	12	0.708	0.794	0.192
PFS	≥75	ExtraTrees	12	0.650	0.762	0.212
PFS	≥75	XGBoost	12	0.550	0.500	0.543
PFS	≥75	XGB_AFT	12	NA	0.560	0.543
PFS	≥75	Cox	24	0.704	0.796	0.095
PFS	≥75	Lasso	24	0.720	0.750	0.097
PFS	≥75	Ridge	24	0.711	0.771	0.094
PFS	≥75	Enet	24	NA	0.754	0.097
PFS	≥75	RSF	24	0.641	0.654	0.111
PFS	≥75	GBM	24	0.708	0.708	0.116
PFS	≥75	ExtraTrees	24	0.650	0.733	0.109
PFS	≥75	XGBoost	24	0.591	0.500	0.870
PFS	≥75	XGB_AFT	24	NA	0.544	0.870
PFS	≥75	Cox	36	0.680	0.802	0.058
PFS	≥75	Lasso	36	0.720	0.738	0.068
PFS	≥75	Ridge	36	0.711	0.786	0.066
PFS	≥75	Enet	36	NA	0.746	0.069
PFS	≥75	RSF	36	0.620	0.579	0.080
PFS	≥75	GBM	36	0.708	0.627	0.085
PFS	≥75	ExtraTrees	36	0.650	0.635	0.082
PFS	≥75	XGBoost	36	0.577	0.500	0.900
PFS	≥75	XGB_AFT	36	NA	0.492	0.913

Note: Values are reported from exploratory age-stratified analyses. Cells shaded in light gray indicate the model with the highest mean C-index within each age stratum for

the corresponding endpoint. Missing C-index values are reported as NA where no valid estimate was available. OS, overall survival; PFS, progression-free survival; IBS, integrated Brier score.

Supplementary Table 3 Detailed risk-group assignment and threshold outputs.

Panel A. Threshold summary and prognostic separation

Endpoint	Selected model	Time threshold (months)	Optimal predicted-risk cutoff	Comparison	Hazard ratio (95% CI)	P value
OS	Ridge	24	0.6784	High vs Low	4.57 (2.81–7.43)	<0.001
PFS	Ridge	12	0.3943	High vs Low	3.89 (2.47–6.14)	<0.001

Panel B. Distribution of model-derived risk groups in the testing cohort

Endpoint	Grouping scheme	Assigned group	No. of patients	No. of events	Events/Total
OS	Binary risk group	Low	42	32	32/42
OS	Binary risk group	High	52	51	51/52
OS	Tertile risk group	Low	32	25	25/32
OS	Tertile risk group	Intermediate	31	27	27/31
OS	Tertile risk group	High	31	31	31/31
PFS	Binary risk group	Low	44	36	36/44
PFS	Binary risk group	High	50	50	50/50
PFS	Tertile risk group	Low	32	26	26/32
PFS	Tertile risk group	Intermediate	31	29	29/31
PFS	Tertile risk group	High	31	31	31/31

Note: Threshold outputs were derived from Ridge-based models for consistency and interpretability in exploratory risk-threshold analysis across endpoints. The binary classification was based on the optimal predicted-risk cutoff, whereas tertile classification was based on the distribution of model-derived predicted risk. Hazard ratios compare the high-risk and low-risk groups from the binary classification. All assignments were generated in the independent testing cohort.
